# Impulsivity, suicidal thoughts, psychological distress, and religiosity in adolescents and young adults

**DOI:** 10.3389/fpsyt.2023.1137651

**Published:** 2023-04-05

**Authors:** Mudassar Abdullah, Muhammad Tahir Khalily, Anthony Charles Ruocco, Brian Hallahan

**Affiliations:** ^1^Department of Psychology, International Islamic University, Islamabad, Islamabad, Pakistan; ^2^Department of Clinical Psychology, Faculty of Social Sciences and Humanities, Shifa Tameer-e-Millat University, Islamabad, Pakistan; ^3^Department of Psychological Clinical Science, University of Toronto, Toronto, ON, Canada; ^4^Department of Psychiatry, University of Galway, Galway, County Galway, Ireland

**Keywords:** impulsivity, suicidal ideation, distress, religious commitment, spirituality

## Abstract

**Background:**

Impulsivity is associated with suicidal acts and ideation, whereas higher religious commitment has been identified as a potential protective factor linked to lower suicidal ideation.

**Objectives:**

We examined the extent to which higher religious commitment is associated with lower suicidal ideation and whether religious commitment modifies the relationship between impulsivity and suicidal ideation.

**Methods:**

Adolescent and young adult males, with a prior history of suicidal act and ideations, completed standardized questionnaires [i.e., Beck Scale for Suicidal Ideation (BSS), Barratt Impulsivity Scale-II (BIS-II), Depression Anxiety Stress Scale (DASS), and Religious Commitment Inventory-10 (RCI-10)], to assess impulsivity, suicidal ideation, distress, and religious commitment. Regression and mediation analyses were performed to investigate the relationships among impulsivity, religious commitment, and suicidal ideation.

**Results:**

Of the 747 study participants (mean age 18.8 years, SD = 4.1), 151 (20.2%) had a history of suicidal acts and 177 (23.7%) had a history of suicidal ideation. Non-planning impulsivity (predictor) was inversely associated with religious commitment (*r* = −0.33, *p* < 0.01), and religious commitment (mediator) was inversely related to suicidal ideation (outcome) (*r* = −0.32, *p* < 0.01). These findings remained statistically significant when controlling for either religious commitment or non-planning impulsivity, as appropriate. Higher religious commitment reduced the association between non-planning impulsivity and suicidal ideation (*p* < 0.01).

**Conclusion:**

The findings highlight the potential for cultivating spirituality to buffer against higher suicidal ideation, and thus could be considered as an additional therapeutic strategy for individuals with higher levels of impulsivity and co-morbid suicidal ideation.

## Introduction

Suicide is the leading cause of mortality in adolescents and young adults worldwide. Approximately 9 individuals per 100,000 die by probable suicide on an annual basis with higher levels noted in those aged 18–35 years, with suicide consequently the fourth highest cause of mortality in this age group (1). Suicide rates in Pakistan are consistent with these global figures, with particularly high rates noted in young males (standardized suicide rate of 21.9 per 100,000). In Pakistan, it has been estimated that approximately 19,311 suicides occurred in 2019, with rates approximately 3 times higher in males compared to females (14,771 v. 4,560) (1), with exact rates difficult to estimate due to non-attendance in medical facilities after episodes of suicidal act with legal, religious and social stigma pertaining to acts of suicide particularly prevalent.

Both suicidal act and suicidal ideation have been associated with a large array of demographic and clinical factors. Demographic factors include younger age (2), female gender (3), lower educational or vocational attainment (4), and childhood trauma (5). Clinical factors include the presence of psycho-active substance use (6), borderline or antisocial personality disorders (7), other mental health disorders including mood, anxiety and psychotic disorders (8), and previous suicidal act or suicidal ideation (9).

Higher rates of suicidal act and suicidal ideation have been consistently observed in individuals with greater levels of impulsivity (10). Impulsivity, however, is not a singular construct and includes attentional, motoric, and reflection or non-planning components (11, 12). Previous work has suggested that individuals with a past history of suicidal act and suicidal ideation often score higher on attentional and motoric impulsivity measures (13, 14).

The role of religious commitment—or the degree to which a person adheres to their religious values, beliefs, and practices, and utilizes these in daily living (15)—in modifying impulsivity and buffering its relationship with suicidal ideation is largely unexplored. Several studies have shown that greater religious commitment (or beliefs) is associated with lower rates of suicide (16, 17), with some (albeit lesser) evidence of a similar association with suicidal ideation and suicidal act (18, 19); however, these findings have not been consistently observed (20–22). The presence of core religious beliefs has additionally been associated with lower distress for individuals experiencing suicidal ideation [i.e., commitment theory; (23)].

### Rationale

As indicated in our literature, religious beliefs play an important role in reducing suicidal ideation when associated with psychological distress and other cohorts (24). Research on the relationship of suicidal thoughts and behavior, and psychological distress have been consistent (25). Therefore, we aimed to investigate how religious commitment mediates the relationship between impulsivity and suicidal ideation. However, this domain has never before been tested in research settings.

**Figure 1:**
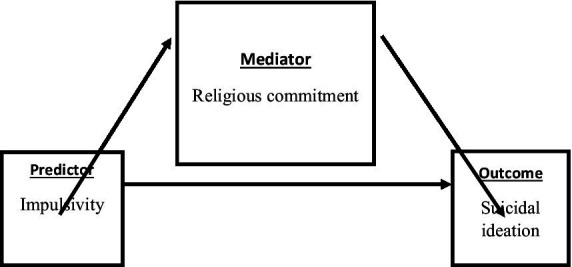
Mediation model.

In the present study, we studied a cohort of adolescents and young men to determine the extent to which higher religious commitment is associated with lower suicidal ideation. We additionally examined if religious commitment mediates the relationship between impulsivity and suicidal ideation (Figure 1).

#### Conceptual framework

Conceptual framework of the study.

## Methods

### Design and participants

Male students (*n* = 747) from nine educational institutions (high school and madrassa, the latter referring to a high school institution with additional religious education in Islam) aged 12–26 years in the Peshawar region of Khyber Pakhtunkhwa in Pakistan were screened for the study. Written informed consent was obtained for individuals 18 years of age and over, with written parental consent and participant assent obtained for those aged 12–17 years. Ethical approval was attained from the Departmental ethics committee (Board of Advance Studies and Research, BASR) at the International Islamic University, Islamabad (F. No. IIU/2021-Exams-6,511).

Basic demographic data were collected through interviews, included age, socio-economic status, and educational level. Clinical data relating to personal and family psychiatric history were also gathered. Reports of suicidal act and ideations were assessed *via* a collateral history (e.g., from parents). The following questionnaires assessing impulsivity, suicidal ideation, religious commitment, and depression, anxiety and stress, were translated (where necessary) into Urdu:

#### Barratt impulsiveness scale

The Barratt impulsiveness scale (BIS-II) consists of 30 items that describe attentional, motoric, and non-planning impulsivity (26), with each item measured on a 4-point Likert scale ranging from *rarely/never* to *almost/always*. A revised Urdu translation of the BIS-II was used in the present study. The reliability for each scale was as follows: *α* = 0.71 (total score), *α* = 0.53 (attentional), *α* = 0.54 (motor), and *α* = 0.63 (non-planning). Previous data have demonstrated a Cronbach’s α for the Urdu translation of the total scale of 0.83 (27).

#### Beck scale for suicidal ideation

The Beck Scale for Suicidal Ideation (BSS) scale consists of 19 items and measures severity of suicidal ideation for each item on a 3-point scale from least to most severe (28). The adapted and translated Urdu version of this instrument has a Cronbach’s *α* = 0.75 (29).

### Religious commitment inventory

The religious commitment inventory (RCI-10) is a 10 item screening instrument to assess the level of one’s religious commitment utilizing a 5-point Likert scale ranging from *Not at all true of me* to *Totally true of me* (15). The RCI-10 evaluates the extent to which an individual adheres to their religious beliefs, values, and practices, and their utilization of these constructs, and consists of six intrapersonal religious commitment items and four interpersonal commitment items. The adapted and translated Urdu version of this instrument has a Cronbach’s *α* = 0.78, with previous data noting a Cronbach’s α of 0.85–0.98 in the English version of this instrument (15).

#### Depression anxiety stress scale

The depression anxiety stress scale (DASS)-42 consists of a set of three self-reported measures (depression, anxiety, and stress) of 14 items each that are rated on a 4-point Likert scale reflecting symptom severity (30). The adapted and translated Urdu version of this instrument has a Cronbach’s *α* = 0.92, with previous data demonstrating Cronbach’s *α* of 0.84–0.97 (31).

The Cronbach’s α were derived from the study sample, and were cross-culturally validated in Urdu for this study. Participants were randomly selected from high school and madrassa settings. Scales were distributed in an extended class room session after initial screening. Each participant answered and returned the questionnaire in a one-hour period. This study was conducted during the routine timings of educational institutions. Appropriate approval was obtained from Heads of the Institutes. Data confidentiality was ensured for preventative purposes.

### Statistical analysis

Statistical analysis was conducted using the Statistical Package for Social Sciences for Windows (SPSS Inc., IBM, New York, United States) Version 23.0. Descriptive statistics included frequency, percentages, mean, and standard deviations. Regression analysis was undertaken to examine the relationships between impulsivity and suicidal ideation with significance set at *p* < 0.01. In Step 1, Psychological distress (measured with DASS) and its subcomponents: depression, anxiety, and stress, in step 2, impulsivity and its subcomponents: attentional, motor, and non-planning, and in step 3, religious commitment were entered. Mediation using ordinary least square path analysis was conducted with the PROCESS macro version 4.2 (32), which was used to examine the effect of impulsivity on suicidal ideation and religious commitment while controlling for age. A bias-corrected bootstrap confidence interval was utilized to examine impulsivity on 10,000 bootstrap samples.

## Results

The demographic and clinical characteristics of the 747 participants are presented in Table 1. The response rate was 74.9% (747 out of 997 invited individuals). Of note, the mean age of participants was 18.8 years (SD = 4.1), with 151 (20.2%) individuals having a prior history of suicidal acts and 177 (23.7%) individuals having a prior history of suicidal ideation, including other characteristics such as attendees of high school 450 (60.2%) and madrassa 297 (39.8%), having socioeconomic status; middle (43.2%) and lower income class (37.8), and domiciliary arrangements; joint family system (61%). Participants’ scores on the BSS, BIS-11, and DASS are also displayed in Table 1.

**Table 1:** Demographic and clinical characteristics.

	*n*	%
Site of education		
Second level school	450	60.2
Third level madrasa	297	39.8
Socio-economic status		
I (upper income class)	142	19.0
II (middle income class)	323	43.2
III (lower income class)	282	37.8
Domiciliary arrangement		
Nuclear family system	291	39.0
Joint family system	456	61.0
History of suicidal act	151	20.2
History of suicidal ideation	177	23.7
	Mean	SD
Age	18.8	4.1
BIS-II		
Total	79.9	9.6
Motoric	31.2	4.4
Attentional	23.4	3.6
Non-Planning	25.4	5.3
BSS	8.6	8.2
RCI-10	35.6	7.5

Table 2 displays the correlations among suicide-related characteristics scores on self-report questionnaires. Suicidal ideation was significantly positively associated with psychopathology symptoms (depression, anxiety, stress), as well as total impulsivity (*r* = 0.17, *p* < 0.01) and non-planning impulsivity (*r* = 0.25, *p* < 0.01). Suicidal ideation was also inversely associated with religious commitment (*r* = −0.32, *p* < 0.01). Additionally, non-planning impulsivity was inversely significantly correlated with religious commitment (*r* = −0.33, *p* < 0.01) (see Table 2).

**Table 2:** Correlation matrix.

Variable	*M*	SD	1	2	3	4	5	6	7	8	9
1. Suicidal ideation	8.6	8.2	–	–	–	–	–	–	–	–	–
2. Psychological distress	41.7	20.1	0.21**	0.91**	0.77**	0.67**	0.01	0.76**	0.66**	0.14**	−0.33**
3. Depression	12.8	8.3	0.22**	0.91**	0.64**	0.03	0.02	0.78**	0.17**	−0.03	
4. Anxiety	12.9	7.3	0.21**	0.86**	0.03	0.03	−0.02	0.68**	−0.01		
5. Stress	16.1	7.1	0.12**	0.03	0.06	0.02	0.02	−0.17			
6. Total Impulsivity	79.9	9.6	0.17**	0.04	0.03	0.02	−0.13				
7. Attentional	23.4	3.6	0.05	0.02	−0.09	−0.07					
8. Motor	31.2	4.4	0.08	0.09	−0.05						
9. Non-planning	25.4	5.3	0.25**	−0.08							
10. Religious commitment	35.6	7.5	−0.32**								

**p < 0.01.

In regression analyses, we entered psychological distress and its subcategories (depression, anxiety and stress) in step 1, impulsivity and its subcomponents (attentional, motor and non-planning) in step 2, and religious commitment in step 3. Model 1 indicated that depression was significantly associated with suicidal ideation (*β* = 0.22, *p* = 0.001) and accounted for 4% of the variance. Model 2 indicated that depression (*β* = 0.14, *p* = 0.015) and anxiety (*β* = 0.13, *p* = 0.041) were significantly correlated with suicidal ideation, with these two variables accounting for 6% of the variance. Model 3 indicated that depression (*β* = 0.15, *p* = 0.006), anxiety (*β* = 0.12, *p* = 0.066), and non-planning impulsivity (*β* = 0.39, *p* = 0.001) were significantly associated with suicidal ideation, with these three variables accounting for 12% of the variance. Model 4 indicated that depression (*β* = 0.15, *p* = 0.007), anxiety (*β* = 0.10, *p* = 0.005), non-planning impulsivity (*β* = 0.26, *p* = 0.001), and religious commitment (*β* = −0.28, *p* = 0.001) were significantly related to suicidal ideation, with these four variables accounting for 17% of the variance (see Table 3).

**Table 3:** Regression analyses.

Model	*B* (95% CI)	SE *B*	*β*	*R* ^2^	*F*	*p*
1. Constant	5.77 (4.72, 6.83)	0.54	0.22	0.05	37.9	<0.001
Depression	0.22 (0.15, 0.29)	0.04	0.14	0.06	21.19	<0.015
2. Constant	5.21 (4.02, 6.39)	0.61	0.12	0.12	31.76	<0.041
Depression	0.14 (0.03, 0.25)	0.06	0.15	0.17	37.63	<0.006
Anxiety	0.13 (0.01, 0.26)	0.07	0.10			<0.066
3. Constant	−4.53 (−7.46, −1.59)	1.50	0.25			<0.001
Depression	0.15 (0.05, 0.26)	0.06	0.15			<0.001
Anxiety	0.12 (−0.01, 0.23)	0.07	0.09			<0.005
Non-planning	0.39 (0.28, 0.49)	0.06	0.17			<0.001
4. Constant	8.70 (4.01, 13.34)	2.38	−0.25			<0.001
Depression	0.15 (0.05, 0.25)	0.06				
Anxiety	0.10 (−0.03, 0.21)	0.06				
Non-planning	0.26 (0.15, 0.37)	0.06				
Religious commitment	−0.28 (−0.36, −0.11)	0.04				

*p* ≤ 0.001, *p* ≤ 0.005.

Mediation analysis was used to investigate the hypothesis that religious commitment mediates the effect of non-planning impulsivity on suicidal ideation. Results indicated that non-planning impulsivity was inversely significantly associated with religious commitment, *B* = −0.45, SE = 0.04, 95%CI [−0.55, −0.36], *β* = −0.32, *p* = 0.001, and that religious commitment was inversely related to suicidal ideation, *B* = −0.29, SE = 0.04, 95% CI [−0.36, −0.21], *β* = −0.26, *p* = 0.001. Non-planning impulsivity was significantly associated with suicidal ideation after controlling for the mediator, religious commitment, *B* = 0.25, SE = 0.05, 95%CI [0.14, 0.36], *β* = 0.16, *p* = 0.001, consistent with partial mediation. Approximately 12% of the variance in suicidal ideation was accounted for by the predictors (*R*^2^ = 0.12). The indirect effect was tested using a percentile bootstrap estimation approach with 10,000 samples (33), implemented with the PROCESS macro Version 4.2 (32). These results indicated that the indirect coefficient was significant, *B* = 0.13, SE = 0.02, 95%CI [0.09, 0.18], completely standardized *β* = 0.09. Non-planning impulsivity was associated with suicidal ideation scores that were approximately 0.13 points higher as mediated by religious commitment.

## Discussion

### Major findings

The current study examined the association of religious commitment with both impulsivity and suicidal ideation. The sample comprised adolescent and young adult males, a substantial proportion of whom reported a history of suicidal acts and thoughts. Higher psychological distress, greater impulsivity, and lower religious commitment were associated with suicidal ideation. Importantly, higher religious commitment had a modifying effect on the relationship between impulsivity and suicidal ideation (Table 4).

**Table 4:** Relationship between impulsivity and suicidal ideation.

Predictive effects	Effect	*B*	SE *B*	*β*	*p*	*R*^2^ (95% CI)
Non-planning impulsivity→Suicidal thoughts	Total	0.38	0.05	0.24	<0.001	0.06 (0.27, 0.49)
Non-planning impulsivity→Religious commitment	Direct	0.25	0.05	0.16	<0.001	0.12 (0.14, 0.36)
Non-planning impulsivity→Religious commitment→Suicidal thoughts	Indirect	0.13	0.02	0.08	<0.001	0.08 (0.09, 0.18)

*p* ≤ 0.001.

Consistent with previous research, higher current psychological distress was associated with suicidal ideation (34–36), including aspects of distress including depressive and anxiety symptoms also significantly associated with suicidal ideation (36).

Impulsivity has consistently been associated with a large range of risk-taking behaviors including alcohol and psychoactive substance use (37, 38) and suicidal acts (39); however, recent work has noted, consistent with our findings, an association between impulsivity and suicidal ideation (40, 41). Consequently, given that greater non-planning impulsivity is associated with higher levels of suicidal ideation, it may be clinically important to identify factors that mediate the association between impulsivity and suicidal ideation.

Higher religious commitment had a modest modifying effect on the relationship between impulsivity and suicidal ideation. Previous work has predominantly focused on the role of religious commitment having a prophylactic role on suicide (42) and suicidal acts (43), and to a lesser extent suicidal ideation (44).

### Implications

This study expands on existing data by demonstrating a modifying aspect of religious commitment on the impact of impulsivity relating to suicidal ideation and suggests that religious commitment may not only be prophylactic for suicidal ideation but could also modify the risk of non-planning impulsivity on suicidal ideation.

### Strengths

The study comprised a large cohort of individuals (*n* = 747) from 9 different educational institutions and for the first time investigated if religious commitment mediated the relationship between impulsivity and suicidal ideation.

### Limitations

The sample was restricted solely to males and from Sunni Islam sects and is not necessarily generalizable to other populations. Additional research including females and people with different religious affiliations is needed. The data collected were cross-sectional and mediation could only be tested within this context. Longitudinal research would assist in clarifying the identified mediation effects. The RCI-10 has high Cronbach’s *α* scores for its’ English version but requires further cultural validation for its’ Urdu version. Finally, the age range of the study was limited to adolescents and young adults. Research on individuals across the lifespan would illuminate the extent to which these findings are relevant to children and older adults.

## Conclusion

We demonstrated that greater psychological distress and impulsivity as well as lower religious commitment predicted higher suicidal ideation, with religious commitment having a modifying effect on the relationship between impulsivity and suicidal ideation. This study suggests that cultivating spirituality could serve as a protective factor for reducing suicidal ideation in adolescent and young adult males, and may be an additional suggestion for individuals with high levels of impulsivity and histories of suicidal ideation and self-harm.

## Data availability statement

The original contributions presented in the study are included in the article/Supplementary material, further inquiries can be directed to the corresponding authors.

## Ethics statement

The studies involving human participants were reviewed and approved by Departmental ethics committee (Board of Advance Studies and Research, BASR) at the International Islamic University, Islamabad (F. No. IIU/2021-Exams-6,511). Written informed consent to participate in this study was provided by the participants’ legal guardian/next of kin.

## Author contributions

MA and MK were principally involved in study conception and study design with all authors having input into study design prior to study commencement. MA developed the theoretical framework of this study under the supervision of MK. MA was involved in data collection, data analysis, and interpretation of results. AR and BH provided support where required for this study and reviewed all aspects of the study including data interpretation and study write up. All authors reviewed and discussed the results and contributed to the final manuscript and approved the study prior to study submission.

## Conflict of interest

The authors declare that the research was conducted in the absence of any commercial or financial relationships that could be construed as a potential conflict of interest.

## Publisher’s note

All claims expressed in this article are solely those of the authors and do not necessarily represent those of their affiliated organizations, or those of the publisher, the editors and the reviewers. Any product that may be evaluated in this article, or claim that may be made by its manufacturer, is not guaranteed or endorsed by the publisher.
