# Supplementary material for: Impulsivity, suicidal thoughts, psychological distress, and religiosity in adolescents and young adults
Source: Front Psychiatry. 2023 Apr 5;14:1137651. doi: 10.3389/fpsyt.2023.1137651 (PMC10113498; doi:10.3389/fpsyt.2023.1137651)
Supplement: Supplementary file 1 [file Data_Sheet_1.pdf]

Scale of Suicidal Ideation

Original references:
Beck AT Kovacs M Weissman A. Assessment of suicidal intention: The scale of suicide ideation. J Consult Clin Psychology. 1979; 47: 343-352.
Beck AT Steer RA Rantieri WF. Scale for suicide ideation: Psychometric properties of a self-report version. J Clin Psychology. 1988; 44: 499-505.

The scale of suicidal ideation consists of 19 items, scored 0 to 2, which can be used to evaluate a patient's suicidal intentions. It can also be used to monitor a patient's response to interventions over time.

Item	Response	Points
1. Wish to live	moderate to strong	0
	weak	1
	none	2
2. Wish to die	none	0
	weak	1
	moderate to strong	2
3. Reasons for living/dying	for living outweigh for dying	0
	about equal	1
	for dying outweigh for living	2
4. Desire to make active suicide attempt	none	0
	weak	1
	moderate to strong	2
5. Passive suicidal desire	would take precautions to save life	0
	would leave life/death to chance	1
	would avoid steps necessary to save or maintain life	2
6. Duration of suicide ideation/wish	brief fleeting periods	0
	longer periods	1
	continuous (chronic) or almost continuous	2
7. Frequency of suicide ideation	rare occasional	0
	intermittent	1
	persistent or continuous	2
8. Attitude toward ideation/wish	rejecting	0
	ambivalent indifferent	1
	accepting	2

Scale of Suicidal Ideation

Item	Response	Points
9. Control over suicidal action/acting-out wish	has sense of control	0
	unsure of control	1
	has no sense of control	2
10. Deterrents to active attempt	would not attempt because of a deterrent	0
	some concern about deterrents	1
	minimal or no concern about deterrents	2
11. Reason for contemplated attempt	to manipulate the environment; get attention or revenge	0
	combination of desire to manipulate and to escape	1
	escape surcease solve problems	2
12. Method: specificity or planning of contemplated attempt	not considered	0
	considered but details not worked out	1
	details worked out and well-formulated	2
13. Method: availability or opportunity for contemplated attempt	method not available or no opportunity	0
	method would take time or effort; opportunity not readily available	1
	method and opportunity available	2
	future opportunity or availability of method anticipated	2
14. Sense of "capability" to carry out attempt	no courage too weak afraid incompetent	0
	unsure of courage or competence	1
	sure of competence courage	2
15. Expectancy/anticipation of actual attempt	no	0
	uncertain not sure	1
	yes	2
16. Actual preparation for contemplated attempt	none	0

Print this page

Scale of Suicidal Ideation

Item	Response	Points
17. Suicide note	none	0
	started but not completed; only thought about	1
	completed	2
18. Final acts in anticipation of death	none	0
	thought about or made some arrangements	1
	made definite plans or completed arrangements	2
19. Deception or concealment of contemplated suicide	revealed ideas openly	0

Scoring:
The total score for the 19 items is calculated.
Minimum score = 0
Maximum score = 38
Higher scores indicate greater suicidal ideation

Close this window

obtained from http://www.psy-world.com
