# Supplementary material for: Impulsivity, suicidal thoughts, psychological distress, and religiosity in adolescents and young adults
Source: Front Psychiatry. 2023 Apr 5;14:1137651. doi: 10.3389/fpsyt.2023.1137651 (PMC10113498; doi:10.3389/fpsyt.2023.1137651)
Supplement: Supplementary file 2 [file Data_Sheet_2.pdf]

DIRECTIONS: People differ in the ways they act and think in different situations. This is a test to measure some of the ways in which you act and think. Read each statement and put an X on the appropriate circle on the right side of this page. Do not spend too much time on any statement. Answer quickly and honestly.

①	②	③	④
Rarely/Never	Occasionally	Often	Almost Always/Always

1	I plan tasks carefully.	①	②	③	④
2	I do things without thinking.	①	②	③	④
3	I make-up my mind quickly.	①	②	③	④
4	I am happy-go-lucky.	①	②	③	④
5	I don’t “pay attention.”	①	②	③	④
6	I have “racing” thoughts.	①	②	③	④
7	I plan trips well ahead of time.	①	②	③	④
8	I am self controlled.	①	②	③	④
9	I concentrate easily.	①	②	③	④
10	I save regularly.	①	②	③	④
11	I “squirm” at plays or lectures.	①	②	③	④
12	I am a careful thinker.	①	②	③	④
13	I plan for job security.	①	②	③	④
14	I say things without thinking.	①	②	③	④
15	I like to think about complex problems.	①	②	③	④
16	I change jobs.	①	②	③	④
17	I act “on impulse.”	①	②	③	④
18	I get easily bored when solving thought problems.	①	②	③	④
19	I act on the spur of the moment.	①	②	③	④
20	I am a steady thinker.	①	②	③	④
21	I change residences.	①	②	③	④
22	I buy things on impulse.	①	②	③	④
23	I can only think about one thing at a time.	①	②	③	④
24	I change hobbies.	①	②	③	④
25	I spend or charge more than I earn.	①	②	③	④
26	I often have extraneous thoughts when thinking.	①	②	③	④
27	I am more interested in the present than the future.	①	②	③	④
28	I am restless at the theater or lectures.	①	②	③	④
29	I like puzzles.	①	②	③	④
30	I am future oriented.	①	②	③	④

Patton, Stanford, Barratt (1995). *J Clin Psy*, vol. 51, pp. 768-774
