# Supplementary material for: Impulsivity, suicidal thoughts, psychological distress, and religiosity in adolescents and young adults
Source: Front Psychiatry. 2023 Apr 5;14:1137651. doi: 10.3389/fpsyt.2023.1137651 (PMC10113498; doi:10.3389/fpsyt.2023.1137651)
Supplement: Supplementary file 3 [file Data_Sheet_3.pdf]

Depression Anxiety and Stress Scale (DASS)

The DASS is a 42-item questionnaire which includes three self-report scales designed to measure the negative emotional states of depression, anxiety and stress. Each of the three scales contains 14 items, divided into subscales of 2-5 items with similar content. The Depression scale assesses dysphoria, hopelessness, devaluation of life, self-deprecation, lack of interest/involvement, anhedonia, and inertia. The Anxiety scale assesses autonomic arousal, skeletal muscle effects, situational anxiety, and subjective experience of anxious affect. The Stress scale (items) is sensitive to levels of chronic non-specific arousal. It assesses difficulty relaxing, nervous arousal, and being easily upset/agitated, irritable/over-reactive and impatient. Respondents are asked to use 4-point severity/frequency scales to rate the extent to which they have experienced each state over the past week.

Scoring:

Scores of Depression, Anxiety and Stress are calculated by summing the scores for the relevant items. The depression scale items are 3, 5, 10, 13, 16, 17, 21, 24, 26, 31, 34, 37, 38, 42. The anxiety scale items are 2, 4, 7, 9, 15, 19, 20, 23, 25, 28, 30, 36, 40, 41. The stress scale items are 1, 6, 8, 11, 12, 14, 18, 22, 27, 29, 32, 33, 35, 39. To use the Scoring Template (below) print on to a plastic overhead. The score for each of the respondents over each of the sub-scales, are then evaluated as per the severity-rating index below.

	Depression	Anxiety	Stress
**Normal**	0 – 9	0 - 7	0 – 14
**Mild**	10 – 13	8 – 9	15 – 18
**Moderate**	14 – 20	10 – 14	19 – 25
**Severe**	21 – 27	15 – 19	26 – 33
**Extremely Severe**	28+	20+	34 +

Norms: Normative data are available on a number of Australian samples. From a sample of 2914 adults the means (and standard deviations) were 6.34 (6.97), 4.7 (4.91), and 10.11 (7.91) for the depression, anxiety, and stress scales, respectively. A clincical sample reported means (and standard deviations) of 10.65 (9.3), 10.90 (8.12), and 21.1 (11.15) for the three measures.

Source: www.psy.unsw.edu.au/groups

Reference : Lovibond, S.H. & Lovibond, P.f. (1995). Manual for the Depression anxiety Stress Scales. (2nd Ed) Sydney: Psychology Foundation.

A centre of excellence supported by the Australian Government

DASS

Name: *Date:*

Please read each statement and circle a number 0, 1, 2 or 3 which indicates how much the statement applied to you *over the past week*. There are no right or wrong answers. Do not spend too much time on any statement.

The rating scale is as follows:

0 Did not apply to me at all
1 Applied to me to some degree, or some of the time
2 Applied to me to a considerable degree, or a good part of time
3 Applied to me very much, or most of the time

1	I found myself getting upset by quite trivial things	0	1	2	3
2	I was aware of dryness of my mouth	0	1	2	3
3	I couldn't seem to experience any positive feeling at all	0	1	2	3
4	I experienced breathing difficulty (eg, excessively rapid breathing, breathlessness in the absence of physical exertion)	0	1	2	3
5	I just couldn't seem to get going	0	1	2	3
6	I tended to over-react to situations	0	1	2	3
7	I had a feeling of shakiness (eg, legs going to give way)	0	1	2	3
8	I found it difficult to relax	0	1	2	3
9	I found myself in situations that made me so anxious I was most relieved when they ended	0	1	2	3
10	I felt that I had nothing to look forward to	0	1	2	3
11	I found myself getting upset rather easily	0	1	2	3
12	I felt that I was using a lot of nervous energy	0	1	2	3
13	I felt sad and depressed	0	1	2	3
14	I found myself getting impatient when I was delayed in any way (eg, lifts, traffic lights, being kept waiting)	0	1	2	3
15	I had a feeling of faintness	0	1	2	3
16	I felt that I had lost interest in just about everything	0	1	2	3
17	I felt I wasn't worth much as a person	0	1	2	3
18	I felt that I was rather touchy	0	1	2	3
19	I perspired noticeably (eg, hands sweaty) in the absence of high temperatures or physical exertion	0	1	2	3
20	I felt scared without any good reason	0	1	2	3
21	I felt that life wasn't worthwhile	0	1	2	3

Reminder of rating scale:

0 Did not apply to me at all
1 Applied to me to some degree, or some of the time
2 Applied to me to a considerable degree, or a good part of time
3 Applied to me very much, or most of the time

22	I found it hard to wind down	0	1	2	3
23	I had difficulty in swallowing	0	1	2	3
24	I couldn't seem to get any enjoyment out of the things I did	0	1	2	3
25	I was aware of the action of my heart in the absence of physical exertion (eg, sense of heart rate increase, heart missing a beat)	0	1	2	3
26	I felt down-hearted and blue	0	1	2	3
27	I found that I was very irritable	0	1	2	3
28	I felt I was close to panic	0	1	2	3
29	I found it hard to calm down after something upset me	0	1	2	3
30	I feared that I would be "thrown" by some trivial but unfamiliar task	0	1	2	3
31	I was unable to become enthusiastic about anything	0	1	2	3
32	I found it difficult to tolerate interruptions to what I was doing	0	1	2	3
33	I was in a state of nervous tension	0	1	2	3
34	I felt I was pretty worthless	0	1	2	3
35	I was intolerant of anything that kept me from getting on with what I was doing	0	1	2	3
36	I felt terrified	0	1	2	3
37	I could see nothing in the future to be hopeful about	0	1	2	3
38	I felt that life was meaningless	0	1	2	3
39	I found myself getting agitated	0	1	2	3
40	I was worried about situations in which I might panic and make a fool of myself	0	1	2	3
41	I experienced trembling (eg, in the hands)	0	1	2	3
42	I found it difficult to work up the initiative to do things	0	1	2	3

DASS Scoring Template

S
A
D
A
D
S
A
S
A
D
S
S
D
S
A
D
D
S
A
A
D
