# Supplementary material for: Impulsivity, suicidal thoughts, psychological distress, and religiosity in adolescents and young adults
Source: Front Psychiatry. 2023 Apr 5;14:1137651. doi: 10.3389/fpsyt.2023.1137651 (PMC10113498; doi:10.3389/fpsyt.2023.1137651)
Supplement: Supplementary file 4 [file Data_Sheet_4.pdf]

Appendix D

RCI-10

Instructions: Read each of the following statements. Using the scale to the right, CIRCLE the response that best describes how true each statement is for you.

Not at all **Somewhat** **Moderately** **Mostly**
Totally
true of me **true of me** **true of me** **true of me**
true of me
1________________ 2________________ 3________________ 4
________________5

1. I often read books and magazines about my faith.	
2. I make financial contributions to my religious organization.	
3. I spend time trying to grow in understanding of my faith.	
4. Religion is especially important to me because it answers many questions about the meaning of life.	
5. My religious beliefs lie behind my whole approach to life.	
6. I enjoy spending time with others of my religious affiliation.	
7. Religious beliefs influence all my dealings in life.	
8. It is important to me to spend periods of time in private religious thought and reflection.	
9. I enjoy working in the activities of my religious affiliation.	
10. I keep well informed about my local religious group and have some influence in its decisions.	
